# Comparison of Clinical Performance of C-MAC Video Laryngoscope Guided vs Blind Placement of I-Gel® in Paediatric Patients: A Randomized Controlled Open-Label Trial

**DOI:** 10.4274/TJAR.2023.221010

**Published:** 2023-08-18

**Authors:** Rakesh Kumar, Pooja Bihani, Sadik Mohammed, Rashmi Syal, Pradeep Bhatia, Rishabh Jaju

**Affiliations:** 1 Department of Anaesthesiology, All India Institute of Medical Sciences (AIIMS), Jodhpur, India; 2 Department of Anaesthesiology, Dr. Sampurnanand Medical College, Jodhpur, India; 3 Department of Anaesthesiology, All India Institute of Medical Sciences (AIIMS), Deoghar, India

**Keywords:** Bronchoscope, children, functional performance, general anaesthesia, supraglottic airway device

## Abstract

**Objective:**

Placement of the supraglottic airway devices under direct vision has been shown to decrease the incidence of malposition in adults. This study was designed to compare the clinical performance of C-MAC guided and blind placement of i-gel® in paediatric patients.

**Methods:**

The present prospective, randomized controlled study was conducted on 102 paediatric patients scheduled to undergo elective infraumbilical surgeries under general anaesthesia. Patients were randomly divided into group “B” (blind) and group “C” (C-MAC) based on the technique used for placement of i-gel®. The primary objective of the study was to compare the incidence of malposition based on the fiberoptic bronchoscope (FOB) score of the glottic view. Oropharyngeal leak pressure (OPLP), hemodynamic parameters, and insertion characteristics (time taken to insert and the number of attempts) were secondary objectives. Categorical data were presented as ratio or percentage and continuous data were presented as mean ± standard deviation or median [95% confidence interval (CI)].

**Results:**

The incidence of malposition (Brimacombe score 1 or 2) was significantly lower in group C compared to group B (7.8% vs 49% respectively) (*P* < 0.001); implying a relative risk reduction of 2.42 (95% CI 1.72 to 3.40) with C-MAC. On FOB assessment, the median (interquartile range) Brimacombe score was significantly better in group C [4 (4-4)] compared to group B [3 (2-3)] (*P* < 0.001). The OPLP was significantly higher in group C compared to group B. Other insertion characteristics were comparable in both the study groups.

**Conclusion:**

Compared to blind placement, C-MAC guided placement ensures proper alignment of i-gel® with periglottic structures and proper functioning of i-gel®.

**Main Points**

• Supraglottic airways (SGA) have become the cornerstone for paediatric airway management. But routinely SGAs are inserted blindly.

• Radiological studies have shown that the incidence of malposition after blind insertion of SGA may exceed more than 50%.

• Malpositioned SGA devices may result in failed insertion, displacement after insertion, multiple attempts at insertion, leak during ventilation, airway trauma, aspiration of gastric contents, postoperative hoarseness, and extubation related problems.

• In this randomized controlled trial, compared to blind insertion, video laryngoscope guided insertion ensures proper alignment of i-gel® with periglottic structures and proper functioning of i-gel®.

## Introduction

The second-generation supraglottic airway (SGA) devices have revolutionized airway management in patients scheduled for surgery under general anaesthesia (GA). Each device possesses some unique features and has been used extensively in paediatric patients with varying degrees of success.^1^ The i-gel® (Intersurgical Ltd, Wokingham, Berkshire, UK), a second-generation SGA, can be placed easily with fewer attempts and provide an effective seal around the airway enabling both spontaneous and controlled ventilation. The blind insertion technique is routinely used to insert the i-gel®. Radiological studies have shown that incidence of malposition after blind insertion of LMA may range up to 50-80%.^2^ A study in paediatric patients undergoing magnetic resonance imaging found that radiologically proven malposition of LMA-unique was seen in 42.9% of children, though the clinical performance of LMA-unique was not hampered.^3^ The consequences of the malpositioned SGA devices include failed insertion, displacement after insertion, multiple attempts at insertion, leak during ventilation, airway trauma, aspiration of gastric contents, postoperative hoarseness, and extubation related problems such as airway edema or laryngospasm.^4^

Video laryngoscope (VL) or other devices to assist under vision placement of SGA devices in adults have been shown to provide optimal placement with higher oropharyngeal leak pressure (OPLP) and better ventilation.^5,6^ However, very few studies have evaluated VL-guided placement of SGA devices in paediatric patients.^7^ Therefore, we planned a study to compare the C-MAC guided i-gel® insertion with that of the blind insertion technique in paediatric patients with regard to correct positioning. We hypothesized that there would be no difference in the incidence of malpositioning of i-gel® between the C-MAC guided and blind insertion technique in paediatric patients. The primary objective of the study was to compare the incidence of malposition of i-gel® between blind and C-MAC guided insertion. Simultaneously, the insertion characteristics such as the number of attempts, ease of insertion, and time required for successful insertion and the OPLP were also compared between two insertion techniques of i-gel® in paediatric population.

## Methods

The present prospective, open-label, randomized controlled trial was conducted in a tertiary care referral institute, after obtaining approval from the Institutional Ethics Committee of All India Institute of Medical Sciences (AIIMS/IEC/2019-20/783) and informed written consent from parents. The study was prospectively registered with the Clinical Trial Registry of India (CTRI: www.ctri.nic.in) (ref. no- CTRI/2019/05/019405; Date of registration- 29/05/2019; Patient enrolment date- 05/06/2019). We recruited 110 children aged between 2 and 6 years, belonging to the American Society of Anesthesiologists (ASA) physical status I or II, and scheduled for elective infra-umbilical surgery in supine position under GA. Children with an anticipated difficult airway, having a history of upper respiratory tract infection, gastroesophageal reflux, mass in the pharyngeal/laryngeal cavity and syndromic babies were excluded from the study.

Enrolled children were randomly assigned in a 1:1 ratio into 2 groups using the block randomization technique. An equal number of blocks of size 4 were used to divide all the patients into two groups (group B and group C). A sealed opaque envelope method was used for allocation concealment and was opened just before shifting the child inside the operating room (OR). Because of the intervention selected it was not possible to practice the double-blinding however, the outcome assessor (bronchoscopy and OPLP measurement) was not aware of the group allocation.

An appropriately lubricated i-gel was inserted blindly in group B and under direct vision using C-MAC (C-MAC® Karl Storz, Tuttlingen, Germany) VL of size 2 MAC blade in group C. The appropriate size of i-gel® was selected as per the manufacturer’s recommendation i.e. size 1.5, 2, and 2.5 for children weighing 5-12, 10-20 and 20-30 kg respectively.

All patients underwent pre-anaesthesia evaluation a day before scheduled surgery and were kept fasted preoperatively according to the Indian Society of Anesthesiologists fasting guidelines.^8^ As per our department protocol, premedication with intravenous (IV) ketamine 0.5 mg kg^-1^ was given in the preoperative area on the day of surgery. In children who were not having IV access preoperatively, oral midazolam syrup 0.5 mg kg^-1^ was given 20 minutes before induction of anaesthesia. Inside the OR, monitoring such as electrocardiography, non-invasive blood pressure, and oxygen saturation were attached and induction of anaesthesia was carried out with IV fentanyl 2 µg kg^-1^ and IV propofol 2.5 mg kg^-1^. In children without having IV access, IV cannula was secured after inhalational induction using sevoflurane. After assessing the adequacy of the bag and mask ventilation, IV atracurium 0.5 mg kg^-1^ was given. After 3 min, the appropriate size i-gel® was inserted using either of two techniques according to group allocation. In both groups, i-gel® was inserted by the anaesthesiologist with having minimum experience of 5 years in anaesthesia field and who have inserted at least 100 i-gel® in paediatric patients.

In group B, the patient was placed in a “sniffing” position and lubricated i-gel® was inserted blindly by keeping it parallel to the chest wall and then glided downwards and backward along the hard palate with a continuous but gentle push until a definitive resistance was felt. In group C, the C-MAC VL blade was inserted in the vallecula and the epiglottis lifted anteriorly under vision on the video monitor screen. The i-gel® was then advanced till the proximal bowl of the i-gel® gets positioned just below the epiglottis and its placement was labeled satisfactory when the tip of the epiglottis is aligned with the tip of the rim of the proximal cuff of the i-gel®.

After insertion, the device was connected to a breathing circuit and correct placement was assured with continuous end-tidal CO_2_ (ETCO_2_) monitoring and the presence of bilateral equal chest rise. When the device was not placed properly on the first attempt, the chin lift followed by chin lift plus jaw thrust maneuvers were used sequentially to correctly place the device. Depending upon the number of maneuvers required to correctly place the i-gel®, the ease of insertion was graded as very easy, easy, or difficult, with no maneuver, one maneuver, and two maneuvers respectively required to place the device.

Anaesthesia was maintained with 1-2% sevoflurane in a mixture of air and oxygen (40:60). Mechanical ventilation was commenced with a tidal volume (V_T_) of 8 mL kg^-1^ and increased to 10 mL kg^-1^ if some leak was encountered. However, if V_T_ of 10 mL kg^-1^ was not delivered, reinsertion was attempted. The respiratory rate was adjusted to maintain an ETCO_2_ of 32-38 mmHg. The OPLP was measured by closing the expiratory valve of the circle system and setting the fresh gas flow to 3 L min and the OPLP was recorded as airway pressure at which equilibrium was reached and an audible leak occurred at the neck. A well-lubricated gastric tube was inserted through the gastric port of the i-gel®. For detection of malposition (alignment with laryngeal opening) of the device, a fiberoptic bronchoscope (FOB) was inserted through the airway tube and placed 0.5 cm proximal to the distal end of the i-gel®. The fibreoptic glottis view was graded using Brimacombe score^9^ (1-vocal cords not seen, 2-vocal cords plus anterior epiglottis seen, 3-vocal cords plus posterior epiglottis seen, and 4-only vocal cords visible). We considered grades 3 and 4 as correct positions while scores 1 and 2 as malposition of i-gel® as per our study protocol.

The insertion time (seconds) was defined as the time from picking up the device to the first appearance of continuous ETCO_2_ tracing on the anaesthesia monitor. The number of attempts to insert the i-gel® was also recorded and a maximum of three attempts were allowed, after which patients were excluded from the study and an alternative device was used to secure the airway.

At the end of the surgery, neuromuscular blockade was reversed with IV glycopyrrolate and neostigmine, and after the return of adequate muscle power; the i-gel® was removed and observed for any blood staining on the device. The child was observed in the post-anaesthesia care unit (PACU) for two hours for any episodes of desaturation, PONV, sore throat, or hoarseness of voice.

### Statistical Analysis

The sample size was calculated using G*Power software (version 3.1.9.2, Institute of Experimental Psychology, Heinrich Heine University, Dusseldorf, Germany).^10^ Prior study indicates that the incidence of malposition using the blind insertion technique ranges from 50-80%.^4^ Assuming an incidence rate of malposition with C-MAC guided technique as 0.5 or 50%, 47 subjects in each group were required at 90% power (1-β) and 5% significance (α) to reject the null hypothesis of equal incidence of malposition for C-MAC and blind insertion technique. We assumed a 10% dropout rate so our sample size was 51 subjects in each group. We used a continuity-corrected chi-squared statistic or Fisher’s exact test to evaluate this null hypothesis.

The recorded data were tabulated in a Microsoft Excel spreadsheet and analyzed using SPSS version 23 (Statistical Package for Social Sciences, Inc., Chicago, IL). The data normality was tested with Kolmogorov-Smirnov one-sample test. Categorical data were presented as a ratio or percentage. Continuous data were expressed as mean ± standard deviation or median [interquartile range (IQR)] (range). The chi-square test or Fisher’s exact test was used to analyze the categorical variables while the intergroup comparison of continuous outcomes was analyzed using an Independent Samples t-test or Mann-Whitney U test. The strength of association between insertion technique and the anatomical fit of the device was calculated in terms of the relative risk reduction. The statistical significance was represented as a confidence interval and the level of significance was set at *P* < 0.05.

## Results

**Figure 1:**
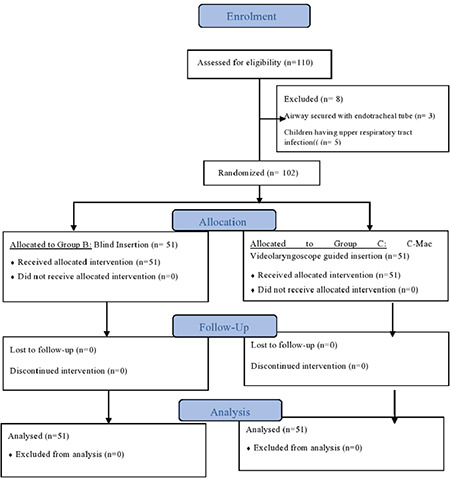
CONSORT flow diagram.

A total of 110 children were assessed for eligibility. Three patients developed desaturation after induction so the airway was secured with an endotracheal tube (ETT) and 5 patients had an upper respiratory tract infection. So, a total of 102 children were finally recruited for the trial and randomized evenly into two treatment groups. Data from both groups were collected and analyzed according to the assigned groups (Figure 1). The demographic characteristics (age, gender, weight, height, and body mass index) and the size of the device used were comparable between both groups (Table 1).

**Figure 2:**
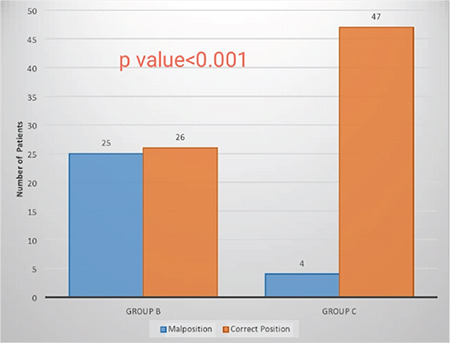
Comparison of malposition of i-gel^®^ between both the groups.

On FOB assessment, the median [(IQR) (range)] of Brimacombe score was significantly better in group C [4 (4-4) (1-4)] compared to group B [3 (2-3) (1-4)] (p≤0.001).The incidence of malposition (Brimacombe score 1 or 2) was significantly lower in group C compared to group B (7.8% vs 49% respectively) (*P *≤ 0.001); implying a relative risk reduction of 2.42 (95% CI 1.72 to 3.40) with C-MAC (Figure 2).

The insertion time (sec) was significantly higher in group C compared to group B [mean difference (95% CI) -7.1 (-8.7 to -5.5); *P *≤ 0.001] (Table 2). The OPLP was also significantly higher in group C compared to group B [mean difference (95% CI) -4.3 (-4.9 to -3.6); *P *≤ 0.001] (Table 2). The first attempt success rate was significantly higher in group C compared to group B (44 vs 35) (*P*=0.033). The ease of device insertion as calculated from the no of maneuvers required for correct positioning of the device was more in group C than in group B. The median [(IQR) (range)] of maneuvers required for correct positioning in groups B and C was 2 [(1-2) (1-3)] and 1 [(1-2) (1-2)] respectively with a median difference (95% CI) 1.0 (0.05 to 0.5); *P *≤ 0.018 (Table 2).

In group B, 4 patients while in group C one patient had blood on the device after removal. One patient in group B had a minor dental injury (Table 3). None of the children in either group experienced hoarseness of voice or sore throat.

## Discussion

The present study found that the C-MAC guided insertion of i-gel® was associated with a significantly lower incidence of malposition and significantly higher OPLP compared to the blind insertion technique. Also, C-MAC guided insertion of i-gel® was associated with significantly better first-attempt success rate and ease of device insertion, and lower device-related adverse effects.

The advancement in anaesthesia practice is towards performing procedures under the vision and includes ultrasound assistance for regional blocks, ultrasound-guided central venous cannulation, fiberoptic/VL guided endotracheal intubation, etc. Anaesthesiologists almost confirm the correct position of the ETT and corrective measures are immediately taken in the context of misplaced ETT but often accept suboptimally placed SGAs.^11^ The Difficult Airway Society and the ASA difficult airway guidelines recommend blind airway management unreliable and VL has become an integral part of airway management.^12,13^ The correct placement of SGAs after blind insertion is often assessed by indirect measures such as adequate chest rise, ETCO_2_ monitoring, measurement of OPLP, leaks during ventilation and cuff pressure. The fiberoptic assessment of the glottis view provides the most reliable assessment of the correct position of SGA but is not practiced routinely. VL offers better glottis visualization on the screen and enables correct placement of SGA beneath the glottis, thereby preventing epiglottic down folding or distal cuff displacement and improving functional or anatomical optimization of SGA.^14^

Fiberoptic evaluation after blind insertion of SGAs has shown the tip of the epiglottis in the bowl of SGA in over 50% of patients.^3^ Other suboptimal positions described are epiglottis downfolding during device insertion, misalignment between the epiglottis and SGA cuff, inappropriate intra cuff pressure or epiglottis obstructing the airway in the bowl of SGA.^2^ In adults, several adjuncts such as Macintosh laryngoscopes, lightwand, C-MAC VL and other VL have been used to guide under vision placement of SGA.^5,6,14,15,16,17,18^

In our study, we observed a 2.42 times risk reduction of malpositioned i-gel® in group C compared to group B in pediatric patients. Behera et al.^7^ evaluated the effectiveness of under vision placement (direct laryngoscopy or VL) of Ambu AuraGain in paediatric patients. The incidence of malposition was 44% in blind insertion, 48% in DL group, and 64% in the VL group with no statistical difference. In fact, the reported incidence of malposition in vision-guided group was higher compared to previous studies as they considered the only laryngeal view without the epiglottis as the optimal position of the SGA.^6^ In paediatric patients, the difficulty might be encountered in lifting the large-sized epiglottis which might be caught in the bowl of Ambu AuraGain. They also found no impact on ventilation and the absence of leaks in the majority of the patients in all three groups.

VL offers distinct advantages over the standard Macintosh blade for LMA insertion. First, the camera on the VL blade offers a wider 60° angle of view compared to just a 15° angle of view with a standard laryngoscope blade. Second, due to the proximity of the camera and light source to the tip of the VL, the glottis can be visualized in proximity allowing optimal insertion and correction of any malposition of the SGA. Third, others can also visualize the screen simultaneously and may help in maneuvers for correcting any suboptimal placement of the device.^18^ Other direct viewing methods such as visual stylet-guided insertion of SGA also allow visual confirmation of positioning of SGA and allow better placement compared to conventional blind technique.^19^ In fact, Van Zundert et al.^20^ proposed the development of a SGA which is to be equipped with cameras and fiberoptic illumination to provide under vision device insertion to enable correct placement of SAD position and to take corrective measures immediately if required.

Apart from bronchoscopic visualization of glottis view, measurement of OPLP is another method for evaluation of functional performance of SGA and defining the seal of the device around the airway. Similar to our results, under vision placement of SGA has reported a higher OPLP compared to blind insertion in adults.^4,14,15,16,17,18^ Under vision optimal placement, cuff inflation to a recommended pressure of 60 cmH_2_O, and immediate corrective measures result in a better seal of i-gel® and higher OPLP. Behera et al.^7^ reported comparable OPLP in all the three groups, blind/DL and VL guided group as they didn’t calculate the exact values of OPLP rather, noted the number of patients who had an audible leak in the mouth at 20 cmH_2_O in each group.

Under vision placement of SGAs has shown a higher success rate over conventional blind insertion.^14,15,16,17,18^ As for blind insertion, we rely on the manufacturer’s recommendation for correct sized device selection, so many times the device had to be replaced by a larger-than-recommended size or a smaller size due to ventilation failure resulting in multiple attempts at insertion or sometimes securing the airway with an ETT. The reason for a better first attempt success rate with VL is that the VL blade displaces the tongue laterally and lifts the epiglottis making a room for i-gel® insertion so it prevents any epiglottic down folding during placement of the device and maneuvers like chin lift/head tilt/jaw thrust can be given by the assistant by directly visualizing the screen.

The time taken to insert the device was more in the vision-guided group as more time was required to do the laryngoscopy, making a room for insertion of the i-gel®, lifting up the epiglottis, and then correctly positioning the device. However, from the clinical point of view, this long time is not important.

In the present study, we highlight the importance of under vision placement of SGA compared to blind insertion for optimal sealing conditions. However, there are a few limitations of the present study. First, all the device insertions were carried out by experienced anaesthesiologists for better generalization of the study result. A learning curve and practice is essential for skill acquisition and better hand-eye coordination during the operation of C-MAC VL. But practice is essential for any procedures so we recommend under vision placement of SGA should also be considered during the training of residents or less experienced anaesthesiologists. Second, we selected i-gel®, a second-generation SGA for comparison, studies may be needed to verify the efficacy of VL guided placement with other available SGAs. Third, we enrolled children above the infantile age group so the result of the study cannot be extrapolated to this vulnerable population. Finally, babies with difficult airway or syndromic babies were excluded from the trial for better generalization of the study result, but the vision-guided placement of SGA might be a useful technique in this population. Future studies with larger sample sizes and using other SGAs are required for better validation of our study results.

## Conclusion

C-MAC guided i-gel® insertion prevents or corrects any malposition, offers better sealing characteristics, and improves postoperative pharyngolaryngeal outcomes. We suggest that i-gel® should be inserted under vision using VL to expand the safety of already available second-generation SGAs in paediatric airway management.

## Figures and Tables

**Table 1:**
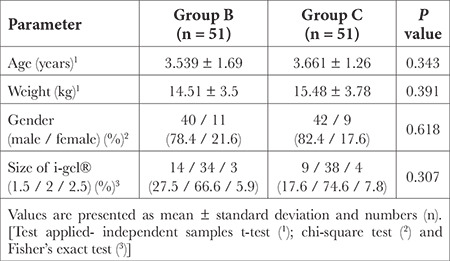
Comparison of Demographic Profile and Size of I-Gel^®^ Used Between Study Groups

**Table 2:**
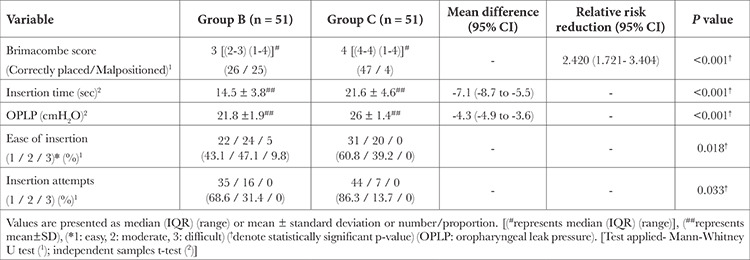
Comparison of Insertion Characteristics and Oropharyngeal Leak Pressure Between Study Groups

**Table 3:**
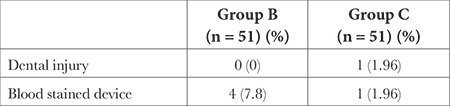
Comparison of Postoperative Complications of I-Gel^®^ Insertion Techniques
